# Effect of exercise on the hypothalamic–pituitary–gonadal axis in a rat model of Alzheimer’s disease

**DOI:** 10.1038/s41598-023-41415-8

**Published:** 2023-08-31

**Authors:** Eman Y. khairy, Ola A. Salama

**Affiliations:** Department of Physiology, Medical Research Institute, Alexandria University, 165, Horreya Avenue, Hadara, Alexandria, Egypt

**Keywords:** Neuroscience, Physiology

## Abstract

Hypothalamic–pituitary–gonadal (HPG) axis dysregulation was suggested to play a crucial role in Alzheimer’s disease (AD). This study investigated the effects of exercise on HPG hormones in an AD rat model, as a possible mechanism underlying the favorable effect of exercise on AD. Forty male Wistar albino rats 2–3 months old were subdivided randomly into two groups (n = 20 each): AD group (injected intraperitoneally with aluminum chloride (70 mg/kg/day) for 6 weeks) and Control group. Each group was subdivided into exercised or non-exercised group (n = 10 each). Exercised groups were subjected to a swimming protocol (60 min/day, 5 days/week, 4 weeks). Serum HPG hormones, hippocampal β-amyloid levels and Morris water-maze cognition were assessed. Results demonstrated higher levels of β-amyloid, gonadotropin releasing hormone (GnRH), luteinizing hormone (LH) and follicle stimulating hormone (FSH) together with lower testosterone levels and cognitive impairment in the AD rats compared to controls. Β-amyloid levels negatively correlated with testosterone levels and positively correlated with GnRH, LH and FSH among the AD rats. Higher testosterone and lower GnRH, LH, FSH and β-amyloid levels, as well as cognitive improvement, were observed in the exercised compared to non-exercised AD rats, suggesting a modulatory role of exercise training on AD-associated HPG axis dysregulation.

## Introduction

Alzheimer’s disease (AD) is a progressive neurodegenerative disorder clinically defined by a gradual decline in cognitive functions that ultimately leads to dementia. The disease process is characterized by damage of neurons and synaptic connections and formation of extracellular amyloid beta (Aβ) plaques and intracellular neurofibrillary tangles, affecting the medial temporal structures, including the entorhinal cortex and the hippocampus, in the earliest stages of the disease then spreads to affect other brain regions^1^.

It has been proposed that the hypothalamus could be both a target of and a contributor to AD pathology^2^. Several studies have demonstrated neuronal loss, neurofibrillary tangles and Aβ plaques in the hypothalamus in AD, suggesting that the hypothalamus is one of the brain areas affected by AD pathology, like the hippocampus and the cortex^3–5^. At the same time, hypothalamic dysfunction has been proposed to be implicated in AD pathogenesis and progression^2,6^.

Moreover, the hypothalamic–pituitary–gonadal (HPG) axis is dysregulated in AD^6^. HPG hormones regulate neuronal development and various brain functions^7^. Receptors of these hormones are located in areas of the brain responsible for memory and learning, such as the hippocampus^7,8^. Additionally, age-related alterations in the HPG hormones have been linked to AD risk^9–12^. Besides, studies using therapies that aimed at modulating the disturbed HPG axis signaling showed a considerable improvement in cognition as well as attenuation in the pathogenesis of AD^13,14^. Taken together, disruption of the HPG axis may have an important role in the AD neuropathology^6^. Therefore, modulation of the HPG axis is a potential therapeutic target in AD.

Physical exercise has been linked to neuroprotection and is regarded as a protective and therapeutic strategy in management of cognitive impairments and neurodegenerative disorders, via different proposed favorable effects on neuronal survival, neuroinflammation, vascularization and brain amyloid burden^15–18^. Besides, early intervention by voluntary exercise has been described to normalize hypothalamic inflammation and neurodegeneration in a mice model of AD^19^. However, little is understood regarding the effects of exercise on the hypothalamic neuroendocrine disruption associated with AD.

Aluminum (Al), a well-known neurotoxin, has been implicated in AD pathogenesis^20^. Aluminum alters the blood brain barrier and precipitates in the brain^21^. Furthermore, Al affects the antioxidant enzyme activity and results in DNA injury of the brain cells and altered brain neurochemistry^22^. Experimentally, long-term exposure to Al has been proved to induce neurodegeneration and neurofilamental modifications in the hippocampus and cerebral cortex associated with impaired cognitive functions^20,23,24^. AlCl_3_-triggered AD in animals is therefore proposed as the animal model that mostly mimics the human AD^25^.

To the best of our knowledge, no studies were conducted for determining the impacts of exercise on the HPG hormones in AD. Therefore, the present study was intended to evaluate the effects of 4 weeks of swimming exercise training on hormones of the HPG axis in an AD rat model and to correlate them with the hippocampal Aβ levels, as a possible physiological mechanism underlying the favorable outcome of exercise on AD.

## Materials and methods

### Experimental animals

Forty adult male Wistar albino rats (aged 2–3 months-old) with initial weight (150–160 g) were included in the study. The animals were purchased from the Animal House of Medical Research Institute, Alexandria University, Egypt. Rats were kept in standard plastic cages (5 animals per cage), fed with standard diet, and water ad libitum. Rats were acclimated to the experimental conditions for at least 2 weeks prior to the study with a 12:12 h light: dark cycle. The experimental procedures in this work were performed in accordance with the National Institutes of Health guide for the care and use of laboratory animals and approved by the Research Ethics Committee of the Medical Research Institute of Alexandria University, Egypt (Approval reference number:AU0122112431). The study was carried out in compliance with the ARRIVE guidelines.

### Experimental design

Animals were subdivided randomly into two groups: an Alzheimer’s disease (AD) group and a non-AD (control) group (n = 20 each). In the AD group, aluminum chloride was injected intraperitoneally (70 mg/kg/day) for 6 weeks according to Ali et al.^26^. AlCl_3_-hydrated (ALCl_3_∙6H_2_O) was obtained from Sigma Chemical Co. (St. Louis, MO, USA) and was freshly dissolved in distilled water. Each group was then subdivided into exercised and non-exercised group (n = 10 each). Rats in the exercised groups were subjected to a swimming exercise program consisted of two phases: adaptation and training, with the training period lasting 4 weeks. After the 4-week exercise program, the learning-memory tests of the rats were performed using the Morris water maze (MWM) test.

### Exercise protocol

The exercised-control group and the exercised-AD group were subjected to a moderate swimming exercise training protocol, in a circular pool (150 cm diameter, 50 cm height), that included 2 phases: adaptation phase and training phase, starting at the beginning of the 3rd week of AD induction. The adaptation phase was used to allow the rats to be adapted to swimming to prevent water-induced stress. During the adaptation phase, the training duration was increased gradually beginning with 15 min on the first day then the exercise time was increased by 15 min each day until animals swam for 60 min on the last day, then the training phase began. During the training phase, animals swam for 60 min/day, 5 days/week for 4 weeks, at water temperature 32 ± 1 °C^27^.

### Assessment of cognitive performance

The Morris water maze test, a hippocampus dependent spatial learning and memory task^28^, was done at the end of the 6th week of AD induction (end of the exercise program). A circular pool (150 cm diameter, 50 cm height), filled with water up to a 35 cm depth, at a temperature of 25 ± 2 °C was used. The maze was divided imaginary into four equal quadrants: Northeast, Northwest, Southeast, and Southwest. A white platform (11 cm diameter) was centered in the Southeast quadrant 1 cm below the water level. Distal visual cues were present throughout the experiment to aid in navigation learning. Liquid milk was added to the water so that the platform was not visible by rats at the water surface. The position of the platform remained unchanged during the training sessions. If rats failed to reach the platform within permitted time, they were gently guided to the platform and placed on it for 15 s. Animals had four training trials per day for 5 days, starting from each of the four different positions in the pool and the time taken to reach the platform (escape latency) during each trial was recorded using stopwatch. Escape latencies for all the daily training trials (four different positions) were averaged per rat then the averages of the groups were calculated. The probe trial was done 24 h after the last acquisition day. The platform was removed and all rats started from the quadrant located opposite to the target quadrant, then the latency to the first target-site crossover was measured to assess reference memory.

### Hippocampal extraction and blood sampling

At the end of the study, blood samples were obtained through cardiac puncture then the rats were sacrificed by cervical dislocation under anesthesia using ketamine/xylazine (100/10 mg/kg). The hippocampus was dissected and washed with physiological saline. Then it was minced and homogenized in phosphate buffer (pH 7.4). The hippocampal homogenates were centrifuged at 5000×g for 5 min and the clear supernatants were collected and stored at – 80 °C after estimation of the protein content by Lowry method^29^. The obtained blood samples were centrifuged and then the separated serum was stored at – 20 °C.

### Biochemical assay

Hippocampal β-amyloid (Aβ) level was determined using a sandwich enzyme-linked immunosorbent assay (ELISA); Rat Aβ1-42 ELISA Kit purchased from Elabscience Biotechnology Inc., Houston, USA, Cat. No. E-EL-R1402. Serum gonadotropin releasing hormone (GnRH) level was measured using ELISA kit purchased from Cloud clone Corp., USA, Cat. No. CEA843Ra. Serum follicle stimulating hormone (FSH) level was estimated using FSH ELISA Kit (Competitive EIA) purchased from LifeSpan Biosciences Inc., WA, USA, Cat. No. LS-F6305. Serum luteinizing hormone (LH) level was measured using LH ELISA Kit (Sandwich ELISA) purchased from LifeSpan Biosciences Inc., WA, USA, Cat. No. LS-F20636. Testosterone level was detected in rat serum using Testosterone ELISA Kit, (ab108666; Abcam, CA, UK). ELISA tests were performed based on the manufacturer’s protocols.

### Statistics

Data were statistically analyzed using IBM SPSS software package version 20.0*.* (Armonk, NY: IBM Corp). The normality of distribution of data was verified using Kolmogorov–Smirnov test. All data had a normal distribution. Data were expressed using mean and standard deviation (SD). F-test (ANOVA) was carried out for comparing between more than two groups followed by post hoc test (Tukey) for pairwise comparison. Paired t-test was used to assess the significance of the difference between escape latencies at the first day and the last day of the acquisition phase within the same group. Pearson’s coefficient test was done to assess the correlation between two normally distributed quantitative variables. Significance of the obtained results was judged at the 5% level.

## Results

### Assessment of spatial learning and memory performance in the MWM

**Figure 1:**
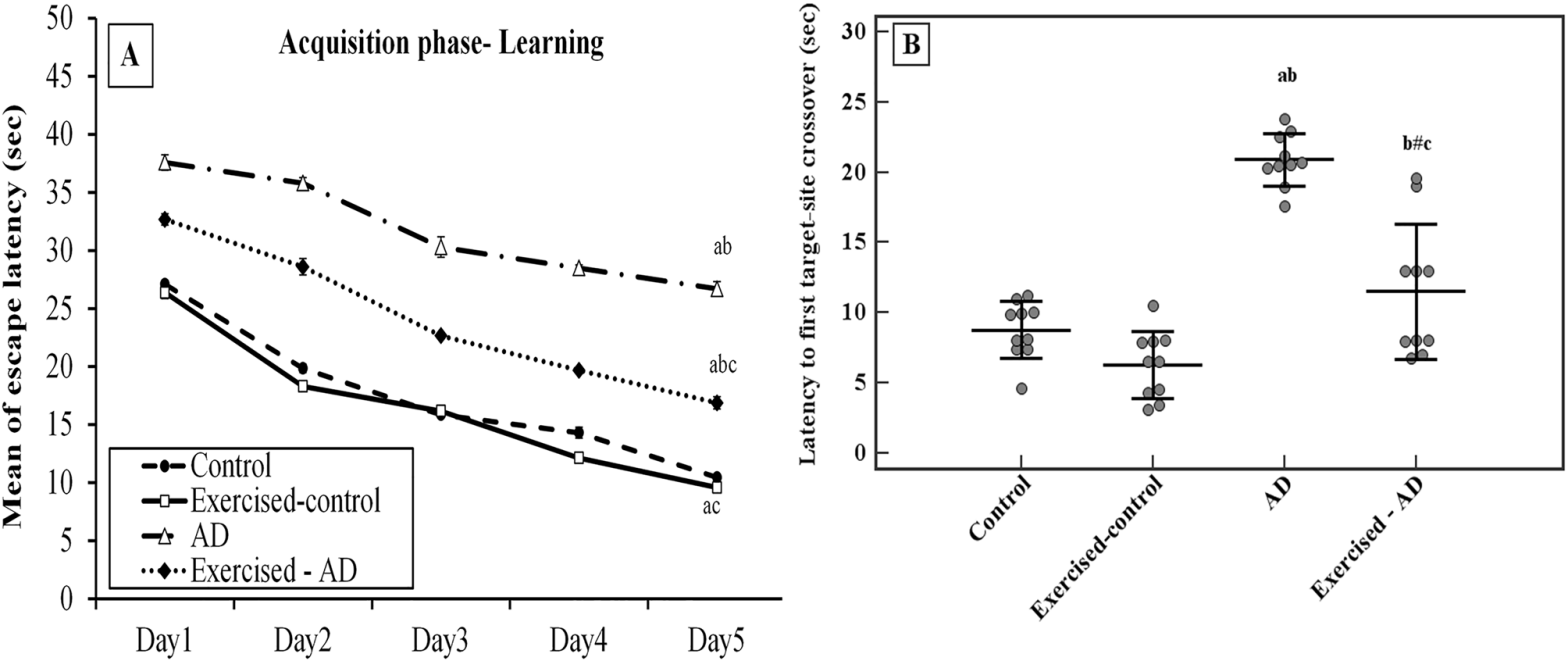
Effect of exercise on cognitive performance in Morris water maze task in all the studied groups (n = 10 each). (**A**): Effect of exercise on learning ability of rats (mean of escape latencies to reach the platform (sec) across days 1–5 of the learning phase). (**B**): Effect of exercise on memory trial of rats (latency to the first target-site crossover (sec) on day 6 during reference memory test, shown as mean ± SD). ANOVA test, post hoc test (Tukey) used for pairwise comparison. AD, Alzheimer’s disease; a: p < 0.001 vs the Control group; b: p < 0.001 vs the Exercised-control group; c: p < 0.001 vs the AD group; b^#^: p = 0.002 vs the Exercised-control group.

As shown in Fig. 1A, the escape latencies decreased throughout the acquisition phase from day one to day five in all groups (p < 0.001 for all groups). Significantly higher mean values of escape latencies were observed in the AD group compared with the control group during all the training days (p < 0.001 for all days). A significant decrease in the mean of escape latency was observed in the exercised-AD group in comparison with the AD group (p < 0.001 for all days).

Latency to the first target-site crossover in the MWM was used to assess reference memory. A significant main effect of AD on the latency to the first target-site crossover was shown (F (1,36) = 82.960, p < 0.001). The rats in the AD group required significantly more time for the first target-site crossover (20.9 ± 1.9 s) when compared with the control group (8.8 ± 2 s), p < 0.001 (Fig. 1B).

However, in the exercised-AD group, the mean of the latencies to the first target-site crossover was significantly lower (11.5 ± 4.8 s) when compared with the AD group (20.9 ± 1.9 s), p < 0.001 (Fig. 1B). Significant interaction effect between AD and exercise training on the latency to the first target-site crossover was found (F (1,36) = 12.908, p = 0.001).

### Hippocampal β amyloid levels

**Figure 2:**
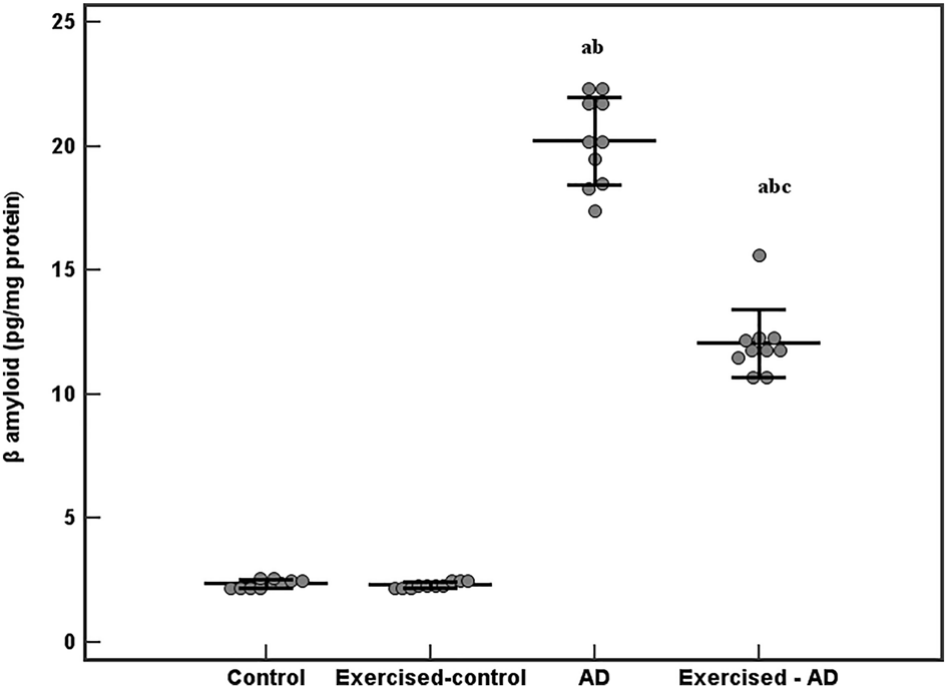
Hippocampal levels of β-amyloid (pg/mg protein) in all the studied groups (n = 10 each). ANOVA test, post hoc test (Tukey) used for pairwise comparison. AD, Alzheimer’s disease; a, p < 0.001 vs the Control group; b, p < 0.001 vs the Exercised-control group; c, p < 0.001 vs the AD group. Data are shown as mean ± SD.

The comparison between hippocampal β-amyloid levels (mean ± SD) in all groups was shown in Fig. 2. Hippocampal β-amyloid levels were significantly higher in the AD group and the exercised-AD group (20.2 ± 1.8, 12.1 ± 1.4 pg/mg protein, respectively) when compared with the control group and the exercised-control group (2.4 ± 0.2, 2.3 ± 0.1 pg/mg protein, respectively), p < 0.001 for all. However, after four weeks of swimming exercise, the level of β-amyloid in the exercised-AD group was significantly decreased as compared to the AD group, p < 0.001. A statistically significant interaction effect was found between AD and exercise training on the hippocampal β-amyloid levels (F (1,36) = 129.666, p < 0.001).

### Assessment of the hypothalamic–pituitary–testicular axis

**Figure 3:**
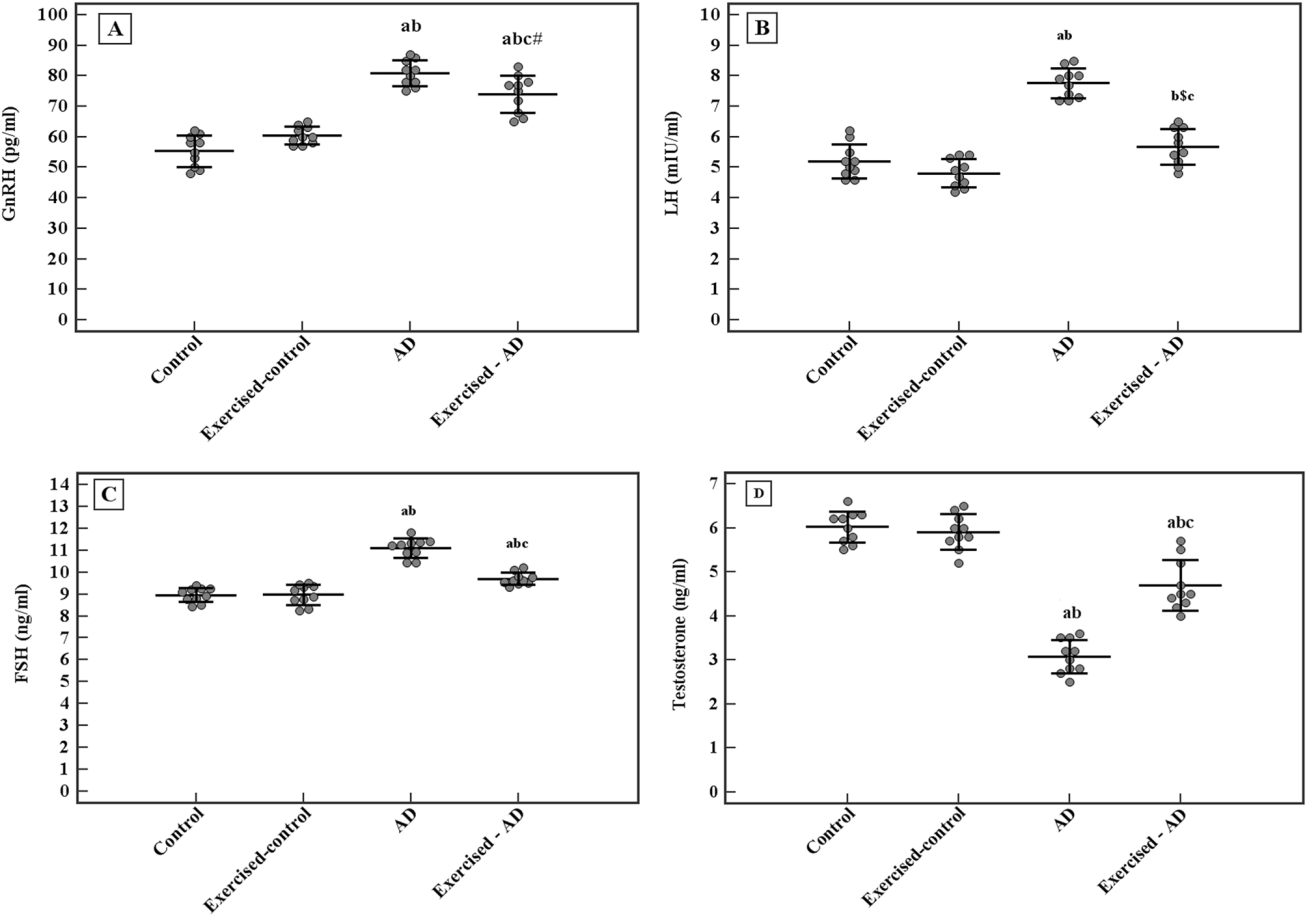
Comparison between the serum levels of the hypothalamic-pituitary–testicular axis hormones in all the studied groups (n = 10 each). (**A**) GnRH, gonadotropin releasing hormone (pg/ml). (**B**) LH, luteinizing hormone (mIU/ml). (**C**) FSH, follicle stimulating hormone (ng/ml). (**D**) Testosterone hormone (ng/ml). ANOVA test, post hoc test (Tukey) used for pairwise comparison. AD, Alzheimer’s disease; a: p ≤ 0.001 vs the Control group; b: p ≤ 0.001 vs the Exercised-control group; c: p ≤ 0.001 vs the AD group; b^$^: p = 0.004 vs the Exercised-control group; c^#^: p = 0.014 vs the AD group. Data shown as mean ± SD.

Data presented in Fig. 3 showed significantly higher serum levels of GnRH, LH and FSH in the AD group (80.9 ± 4.2 pg/ml, 7.8 ± 0.5 mIU/ml, 11.1 ± 0.4 ng/ml, respectively) as compared to the control group (55.4 ± 5.2 pg/ml, 5.2 ± 0.6 mIU/ml, 9 ± 0.3 ng/ml, respectively), p < 0.001 for all. Conversely, serum level of testosterone was significantly lower in the AD group (3.1 ± 0.4 ng/ml) when compared with the control group (6 ± 0.4 ng/ml), p < 0.001. However, 4 weeks of swimming exercise significantly reduced the levels of GnRH, LH and FSH in the exercised-AD group (74.1 ± 6.1 pg/ml, 5.7 ± 0.6 mIU/ml, 9.7 ± 0.3 ng/ml, respectively) as compared to the AD group (80.9 ± 4.2 pg/ml, 7.8 ± 0.5 mIU/ml, 11.1 ± 0.4 ng/ml, respectively), p = 0.014, p < 0.001 and p < 0.001, respectively. Whereas testosterone level was significantly elevated in the exercised-AD group (4.7 ± 0.6 ng/ml) compared to the AD group (3.1 ± 0.4 ng/ml), p < 0.001.

A statistically significant interaction effect was found between AD and exercise training on the levels of the HPG hormones. [(F (1,36) = 15.721, p < 0.001) for GnRH, (F (1,36) = 26.152, p < 0.001) for LH, (F (1,36) = 34. 171, p < 0.001) for FSH, and (F (1,36) = 39.375, p < 0.001) for testosterone].

### Correlation studies

**Figure 4:**
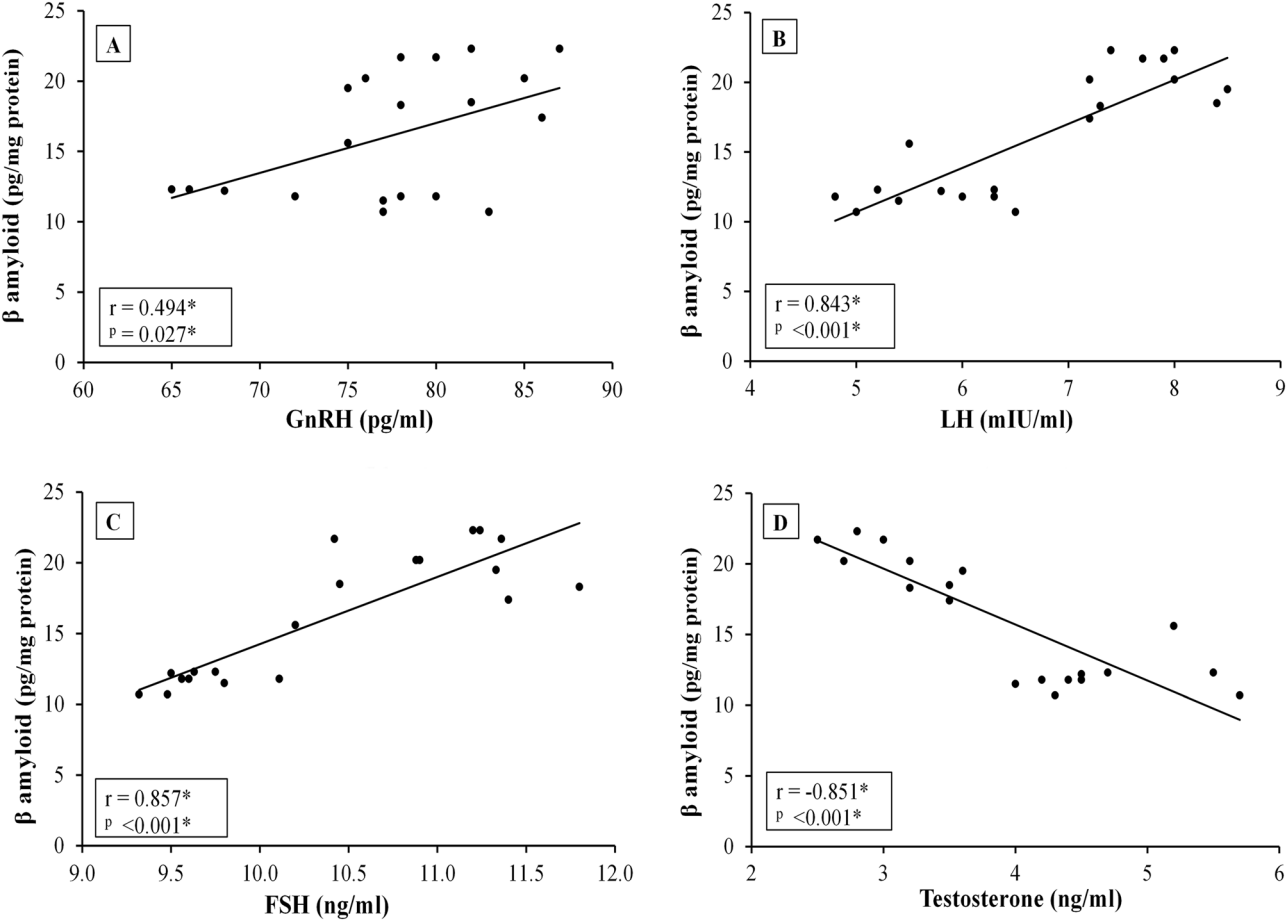
Correlation between hippocampal β-amyloid levels and the hypothalamic-pituitary testicular axis hormones [GnRH, gonadotropin releasing hormone; LH, luteinizing hormone; FSH, follicle stimulating hormone and testosterone] in Alzheimer's disease rats [AD & Exercised-AD] (n = 20). r, Pearson’s coefficient; *, statistical significance at p < 0.05.

In between AD and exercised-AD groups, correlation studies revealed significant positive correlations between the hippocampal β-amyloid level and each of serum GnRH, LH and FSH levels. Whereas a significant negative correlation was detected between hippocampal β-amyloid level and serum testosterone level (Fig. 4). Additionally, the hippocampal β-amyloid level was significantly positively correlated with the latency to the first target-site crossover during reference memory test in the MWM (r = 0.697, p < 0.001).

## Discussion

Several studies have used exercise as a non-pharmacological strategy to slow down or reverse AD^16–18^; however, the detailed mechanisms by which exercise counteracts the negative effects of AD are still not fully understood^19^. Given the well-known role of the HPG axis hormones in several brain functions^7^, the presence of receptors for these hormones in brain areas linked to learning and memory^7,8^, besides the proposed role of the HPG hormonal dysregulation in AD pathophysiology^6^, the present work investigated the impact of exercise on the AD-associated HPG axis hormonal dysregulations as a possible mechanism that may contribute to the explanation of the neuroprotective effects of exercise in AD. Based on the present study, 4 weeks of swimming exercise in AD rats induced HPG axis hormonal modulations that were accompanied with significant reduction in the hippocampal Aβ levels and significant improvement in Morris water-maze cognition compared to non-exercised AD rats.

One of the main pathophysiologic hallmarks in AD is the accumulation of Aβ in the brain owing to exaggerated cleavage of the amyloid precursor protein (APP) by β- and γ- secretases and/or diminished clearance of Aβ in the brain^1^. The deposition of Aβ induces an inflammatory response, which accelerates the disease progression with development of neuroinflammation, oxidative stress, neuronal apoptosis, gliosis and synaptic dysfunction in the AD brain^30^. In the present study, Aβ levels were considerably elevated in the hippocampal tissue of the AD rats compared to the control rats. However, in agreement with the literature^17–19,31^, hippocampal Aβ levels were significantly lower in the exercised compared to the non-exercised AD rats. This was hypothesized to be due to a decrease in Aβ production by activating non-amyloidogenic pathways^18,19,31^. Exercise was found to suppress the unfolded protein response (UPR) and to inhibit the amyloidogenic pathway in the amyloid precursor protein-presenilin 1 (APP/PS1) double-transgenic mice^31^.

An association between the high brain amyloid and cognitive decline is well established in AD^32^. In accordance, in the present study, hippocampal Aβ levels in the AD rats were significantly correlated with cognitive impairment, assessed by measuring the spatial learning and memory, which were significantly impaired in AD rats compared to control rats. Significant improvement in water-maze cognition was observed in the exercised compared to the non-exercised AD rats. Our findings are in agreement with previous studies which demonstrated improvement in cognition with exercise intervention, with several proposed neuroprotective mechanisms, including effects on neuronal survival, neuroinflammation, antioxidant system, vascularization, brain amyloid burden and insulin signaling^15–18,33^.

Hypothalamic dysfunction has been implicated as both a consequence of and a contributor to AD pathology^2–6^. The mechanisms underlying the hypothalamic dysfunction in AD likely include direct impact of Aβ and tau accumulation in the hypothalamus along with the vascular alterations associated with AD. This, in turn, can lead to alterations in the hypothalamic signaling and/or lack of sensitivity to the hormonal feedback signals, leading to alterations in critical physiological functions that possibly exacerbate disease pathology and cognitive decline^2^.

In this regard, dysregulation of the HPG axis hormones has been linked to the neuronal loss that occurs during the pathophysiology of AD^2,6^. Hormones of the HPG axis possess neuronal receptors on different cell types in the limbic system, in particular the pyramidal neurons in the hippocampus which are susceptible to AD pathology. The HPG hormones regulate neuronal development, structure and several functions in the brain^7,8^. During senescence, the HPG axis hormonal dysregulations (elevated GnRH, LH and FSH with reduced sex steroids) promote structural and functional alterations of neuronal cells via different signaling pathways^11^. Altogether, this strongly suggests that modulating the ongoing HPG axis hormonal dysregulation in AD can be used as a potential therapeutic target.

GnRH was considered as a key pathogenic factor in AD^34^, possibly through direct actions on the hippocampal neurons that result in neurodegenerative pathology or indirectly through signaling to increase gonadotropins secretion, particularly LH^2^. Both GnRH and LH have mitogenic properties, therefore it seems plausible that high levels of them may drive differentiated neurons back into the cell cycle, thus triggering nerve cell death and promoting neurodegeneration^35^. Besides, LH promotes the formation of Aβs via activating the amyloidogenic pathways of the APP metabolism such as the β-cleavage of APP by β-secretase enzyme^12–14,36–38^.

In the present study, serum GnRH, FSH and LH levels were significantly higher in the AD rats compared to controls and positively correlated with hippocampal amyloid-β levels. In agreement, marked increases in GnRH expression have been found in the hippocampus of transgenic mice with amyloid pathology^34^. Similarly, a high serum level of LH and FSH has been found in AD patients, this was correlated positively with Aβ levels and impaired cognition^9,12^. Downregulating peripheral LH showed beneficial effects in a clinical trial in AD female patients^39^. Moreover, pharmacological lowering of serum gonadotropin levels was able to save cognitive function in animal models of AD^14,40^ and menopause^41^. This correlated with decreases in the Aβ deposition^14^. LH receptors deficiency in mice with amyloid pathology significantly reduced tau hyperphosphorylation and the Aβ load^42^, hence linking LH to the development and progression of AD.

In the present work, exercise training in AD rats resulted in a significant reduction in serum GnRH, LH and FSH levels compared to non-exercised AD rats. This was associated with improved function in Morris water maze cognitive tasks and reduced Aβ levels. One possible explanation for our findings is the previously reported effect of early exercise intervention on normalizing the inflammation and neurodegeneration in the hypothalamus in AD animal model, suggesting a neuroprotective mechanism that is mediated by the hypothalamus where exercise modulates the hypothalamic dysfunction and hence stops the progression of AD^19^. This modulatory effect may be related directly to the reduction in Aβ burden in the brain. Also, our findings may be the result of a negative feedback associated with the observed elevation in serum testosterone levels in the exercised compared to the non-exercised AD rats. Our results agree with previous studies which reported lower levels of serum gonadotropins after endurance training in both males and females^43,44^. Nevertheless, to the best of our knowledge, no prior studies have examined the effects of exercise on the HPG hormones in AD.

Beside the well-known central effects of Aβ accumulation, it was found to induce deteriorating effects in the periphery^45,46^. It has been recently found that the Leydig cell number is reduced in AD mice^47^. This may explain our observation of a lower serum testosterone level in the AD rats compared to controls. Our finding was in agreement with the previously reported reduced levels of testosterone in men with AD compared with age-matched normal control^48,49^.

In our work, testosterone levels in the AD rats were found to correlate negatively with the hippocampal Aβ levels. In accordance, decreased testosterone levels has been related to a higher risk of AD^12^, with reported improvement in cognition following testosterone treatment^50,51^. Androgen receptors (AR) are expressed in neurons of the hippocampus and amygdala^52^. Testosterone protects the brain against tau protein hyperphosphorylation and Aβ accumulation^53^. Testosterone was reported to reduce the accumulation of Aβ by upregulating the expression of neprilysin; a protease that controls the breakdown of Aβ^54^. Also, it was shown to downregulate β-secretase; the protease that initiates β-cleavage of APP^55^.

Testosterone levels have been reported to increase following acute exercise^56,57^; nevertheless, studies on the impact of chronic exercise on testosterone levels showed conflicting results^44,58–60^. In the present work, significantly higher testosterone levels were found in the exercised compared with the non-exercised AD rats. This is consistent with the previously reported increase in testosterone levels following aerobic exercise programs^58,59^. Also, a study by Sellami et al.^60^ demonstrated that intensive training increased the basal levels of testosterone in middle-aged men and reduced the effect of age on testosterone levels in middle-aged men compared with young men. Exercise was found to enhance testicular function^61^, with observed elevation in Leydig cell number following long-time training in AD mice^47^. Besides, the exercise-induced growth hormone release^62^ may play a role. Growth hormone has been found to have a direct stimulatory effect on the basal secretion of testosterone by rat Leydig cells^63^. Nevertheless, unchanged^64^ or decreased^44^ levels of testosterone have also been reported following exercise programs. This discrepancy may be linked to differences in exercise protocols (duration and intensity of exercise), mode of exercise (endurance versus resistance), age, body mass index and training status of the participants.

## Conclusion

In conclusion, the current study demonstrates a modulatory role for exercise training on the AD-associated HPG axis hormonal dysregulation, that was correlated with reduced β-amyloid levels and cognitive improvement. Our findings point to the importance of the HPG axis as a potential therapeutic target in AD and offer additional evidence for the previously proposed hypothalamic-mediated mechanism by which exercise inhibits the progression of AD^19^. Therefore, this study provides a potential neuroprotective mechanism by which the AD brain benefits from exercise. Further research is required to determine the impact of training variables (type, intensity, duration and frequency), alongside the effect of gender and age on the ability of exercise to modulate the HPG hormones in AD.

## Data Availability

The datasets generated during and/or analyzed during the current study are available from the corresponding author on reasonable request.
